# Rush Medical College

**Published:** 1857-07

**Authors:** 


*Rush Medical College.*

Since the close of the last annual college term, three chairs have been made vacant in this institution, by the resignations of Drs. John Evans, Professor of Obstetrics and Diseases of Women and Children; W. B. Herrick, Professor of Physiology and Pathology; and H. A. Johnson, Professor of Materia Medica and Medical Jurisprudence. Dr. Evans retired from the active practice of his profession two or three years since, and his resignation was but the consummation of a purpose long since formed, and fully understood by his colleagues. The resignation of Professor Herrick was occasioned by such an impaired state of his health, as rendered him unable to perform the active duties of his profession. Both have retired enjoying the full confidence and the most cordial friendship of their colleagues, and we earnestly hope they may yet enjoy many years of prosperity and usefulness. The chair of Obstetrics and Diseases of Women and Children has been filled by the appointment of W. H. Byford, M. D., of Evansville, Indiana. Dr. B. has already acquired a high reputation, both as a practitioner and teacher of medicine, and will bring to the discharge of his duties the qualities of a good writer and speaker, an observing and experienced practitioner, and a ripe scholar. The chair of Physiology and Pathology has been filled by the appointment of H. A. Johnson, M. D., who has just resigned that of Materia Medica, etc. Dr. Johnson having already filled a chair in the college for the last three years, his truly valuable qualities of head and heart are too well known to the friends of the institution to require any comments in this place. John H. Rauch, M. D., of Burlington, Iowa, has been appointed to the chair of Materia Medica, Therapeutics and Medical Jurisprudence, made vacant by the resignation of Dr. Johnson. Dr. Rauch is well known throughout the northwest as a man of high scientific attainments, and is eminently qualified for the place assigned him. With a faculty thus complete in number, and composed of men ardently devoted to the science and practice of the profession, the Rush Medical College will commence its next annual session with every facility for imparting medical knowledge which can be found in our country.

At a recent meeting of the faculty of the college, the following resolutions were prepared and adopted through a committee appointed for that purpose;

*Whereas*, The resignations of Professors W. B. Herrick and John Evans of their respective chairs in Rush Medical College have deprived us, as a faculty, of two of our most experienced and able colleagues—two with whom we have labored and associated on terms the most pleasant and cordial through many years—therefore,

*Resolved*, That we tender to them, in the name of the college, our thanks for their abundant and efficient labors in its behalf; and though compelled to part with them as teachers, we earnestly hope that we may long enjoy their society and counsel as citizens, as friends, as brothers.

*Resolved*, That in the death of Dr. Thomas Spencer, of Philadelphia, late Emeritus Professor of Practical Medicine in Rush Medical College, we have lost a highly esteemed colleague, and the profession one of its most successful, laborious and distinguished members.

